# Alterations in TGF-β signaling leads to high HMGA2 levels potentially through modulation of PJA1/SMAD3 in HCC cells

**DOI:** 10.18632/genesandcancer.199

**Published:** 2020

**Authors:** Kazufumi Ohshiro, Jian Chen, Jigisha Srivastav, Lopa Mishra, Bibhuti Mishra

**Affiliations:** ^1^ Department of Surgery, Center for Translational Medicine, George Washington University, Washington DC, USA; ^2^ Department of Gastroenterology, Hepatology, and Nutrition, The University of Texas MD Anderson Cancer Center, Houston, TX, USA; ^3^ University of Toledo College of Medicine, Toledo, OH, USA; ^4^ Department of Gastroenterology and Hepatology, VA Medical Center, Washington DC, USA

**Keywords:** TGF-β pathway, HMGA2, PJA1, hepatocellular carcinoma

## Abstract

Recently, we observed that the TGF-β pathway is altered in 39% of HCCs. The alterations are correlated with a raised HMGA2 level. Therefore, we compared genetic alterations of HMGA2 and 43 TGF-β pathway core genes in HCC patients from TCGA database. Genetic alterations of 15 genes, including INHBE, INHBC, GDF11, ACVRL and TGFB2 out of 43 core genes, highly-moderately matched that of HMGA2. Co-occurrences of mutation amplification, gains, deletions and high/low mRNA of HMGA2 with those of the core genes were highly significant in INHBE, INHBC, ACVR1B, ACVRL and GDF11. Mass spectrometry studies revealed that HMGA2 interacted with an E3 ligase, PJA1, and that this interaction is enhanced by TGF-β treatment in the nuclear of HCC cells. Co-localization of nuclear PJA1 and HMGA2 in HCC cells increased upon TGF-β treatment. Raised HMGA2 levels that occur with alterations in the TGF-β signaling pathway may reflect an altered activity of E3 ligases, such as PJA1, and potentially contribute to the tumor-promoting roles of TGF-β signaling. Here, we report that the co-occurrence of genetic alterations in HMGA2 and TGF-β pathway core genes is implicated in HCC progression, and propose that HMGA2 and PJA1 may be potential novel targets in dysfunctional TGF-β signaling in HCC.

## INTRODUCTION

The incidence of HCC in the U.S. has increased 3-4 fold in recent years and the overall five-year survival rates remain dismal at 11% [1, 2]. Diagnosis at advanced stages, unclear molecular profiles, and few viable targeted therapeutics are among the factors causing high mortality rates [3, 4]. Moreover, most patients are present with underlying cirrhosis or decompensated liver disease which become difficult to treat with standard doses of chemotherapeutics [5, 6]. Drug resistance is another cause for therapy failure and is associated with the existence of tumor-like stem cells [7-9]. Yet mechanistic insight into pathways driving stem cell transformation, which could lead to targeted therapeutics, is limited for these cancers. Identification and validation of rational targets and signaling pathways underlying HCC development and progression are needed to develop better strategies for HCC diagnosis, prevention, and therapy.

Co-existing cirrhosis and drug resistance from a heterogeneous cancer are some causes for therapy failure: the latter is potentially associated with the existence of cancer stem cells. Therefore, identification and validation of rational targets underlying HCC development and progression- specific populations of HCCs such as those arising from cancer stem cells remain urgently needed for improved survival and decreased toxicities. Recently, we have observed that the TGF-β pathway is altered in 39% of HCCs and that the alterations correlate with raised high mobility group AT-hook 2 (HMGA2) and telomerase reverse transcriptase (TERT) levels [10]. TGF-β pathway members are downregulated in a significant number of human HCCs with a cancer stem cell signature [11, 12]. Additionally, recent TCGA meta-analysis has revealed a significant association between HMGA2 overexpression and poor overall survival in 14 types of cancers, including hepatocellular carcinoma [13]. HMGA2 is a small, non-histone, chromatin associated protein that lacks intrinsic transcriptional activity but regulates gene transcription by altering local chromatin structure at the promoter and/or enhancers [14, 15]. Increased evidence has suggested that HMGA2 could be involved in tumor growth [16-18], cancer cell differentiation [19, 20], and stem cell self-renewal [21, 22].

RING-finger E3 ligases are instrumental in the regulation of inflammatory cascades, apoptosis, and cancer. An increased abundance of negative regulators of the SMAD3-dependent tumor suppression, such as E3 ubiquitin ligases, represent a mechanism for the oncogenic function of the TGF-β pathway. PJA1 belongs to the PRAJA family of E3 ubiquitin ligases, whose expression has been shown to be elevated in some cancers including those of the gastrointestinal tract [23]. Overexpression of PJA1 in HCC cell lines promotes the ubiquitination of SMAD3 and β2SP [23]. PJA1 and SMAD3 respectively bind to SMAD3 and HMGA2. HMGA2 is induced by the TGF-β/SMAD pathway during EMT [24, 25]. To explore targets in HCC in the context of the TGF-β pathway, we analyzed genetic alterations of PJA1 in a significant number of HCCs and determined an interaction between PJA1 and HMGA2.

## RESULTS

**Figure 1:**
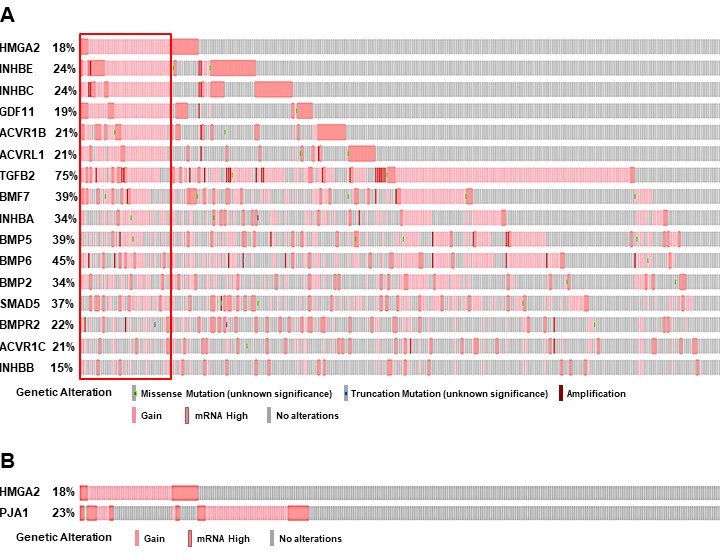
Comparison of amplification, mutations, gain, and high mRNA of HMGA2 and TGF-β pathway core genes. **A**. Amplification, mutations, gain, and high mRNA of HMGA2 were compared with those of 43 TGF-β pathway core genes in 348 HCC patients from TCGA database. Top 15 genes of 43 TGF-β pathway core genes are shown by their genetic alterations that highly-moderately matched with that of HMGA2 (highlighted by red line box). **B**. Gain and high mRNA of HMGA2 were compared with those of PJA1 in 348 HCC patients from TCGA database.

**Figure 2:**
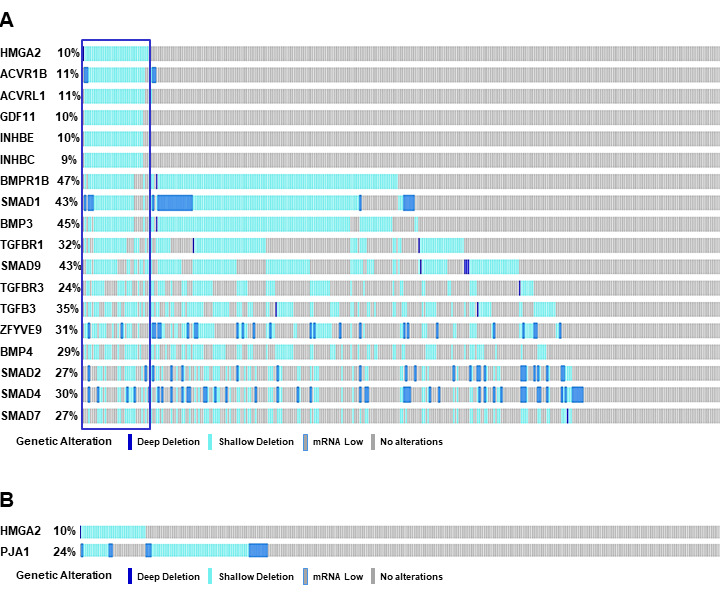
Comparison of deletions and low mRNA of HMGA2 and TGF-β pathway core genes. **A**. Deletions and low mRNA of HMGA2 were compared with those of 43 TGF-β pathway core genes in 372 HCC patients from TCGA database. Top 17 genes of 43 TGF-β pathway core genes are shown by their genetic alterations that highly-moderately matched with those of HMGA2 (highlighted by blue line box). **B**. Deletions and low mRNA of HMGA2 were compared with those of PJA1 in 372 HCC patients from TCGA database.

**Figure 3:**
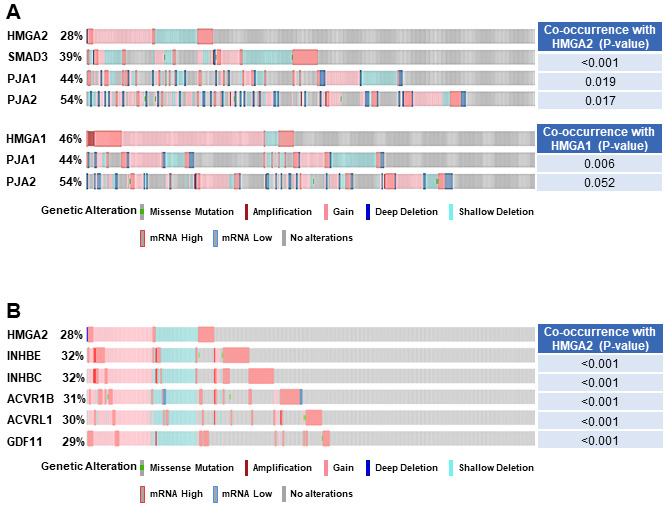
Co-occurrence of genetic alterations of PJA and TGF-β pathway core genes with that of HMGA2. **A**. Co-occurrence of mutation amplification, gain, deletions, and high/low mRNA of HMGA2 and HMGA1 were compared with those of SMAD3 and/or PJA1/2 in 348 HCC patients from TCGA database. **B**. Co-occurrence of mutation amplification, gain, deletions, and high/low mRNA of HMGA2 were compared with those of 43 TGF-β pathway core genes in 348 HCC patients from TCGA database.

Our recent study has demonstrated that HMGA2 was overexpressed in samples with either mutations or amplifications in the TGF-β pathway genes [26]. In addition, the impact on the survival of cancer patients was most significant for overexpression of HMGA2, a collagen-encoding gene and MMP9. Therefore, we compared genetic alterations of HMGA2 and TGF-β pathway core genes in 348 HCC patients from TCGA database. Amplification, mutations, gains, and high mRNA of HMGA2 were compared with those of 43 TGF-β pathway core genes selected in our previous study [26]. Genetic alterations of 15 genes, INHBE, INHBC, GDF11, ACVR1B, ACVRL1, TGFB2, BMP7, INHBA, BMP5, BMP6, BMP2, SMAD5, BMPR2, ACVR1C, and INHBB, of the 43 TGF-β pathway core genes highly-moderately matched that of HMGA2 as highlighted by the red line box (Figure 1A). Although the gain and high mRNA of HMGA2 were compared with those of PJA1 in 348 HCC patients from TCGA analysis data, the genetic alterations lowly matched between the two genes (Figure 1B). Like Figure 1A, deletion and low mRNA of HMGA2 were compared with those of 43 TGF-β pathway core genes in 372 HCC patients from TCGA database. Genetic alterations of 17 genes, ACVR1B, ACVRL1, GDF11, INHBE, INHBC, BMPR1B, SMAD1, BMP3, TGFBR1, SMAD9, TGFBR3, TGFB3, ZFYVE9, BMP4, SMAD2, SMAD4, and SMAD7, of 43 TGF-β pathway core genes were shown to highly-moderately match that of HMGA2 as highlighted by the blue line box (Figure 2A). Deletions and low mRNA in generic alterations of HMGA2 moderately matched that of PJA1 in 372 HCC patients from TCGA database (Figure 2B). Co-occurrences of genetic alterations of PJA1/2 and TGF-β pathway core genes with that of HMGA2 were determined. Co-occurrences of mutation amplification, gain, deletions and high/low mRNA of HMGA2 with those of SMAD3, PJA1, and PJA2 in 348 HCC patients were significant with <0.001, 0.019, and 0.017 of P-values, respectively, while HMGA1 indicated high and low significant co-occurrences with PJA1 and PJA2, respectively (Figure 3A). Co-occurrences of mutation amplification, gain, deletions, and high/low mRNA of HMGA2 with those of 43 TGF-β pathway core genes in 348 HCC patients were highly significant in INHBE, INHBC, ACVR1B, ACVRL and GDF11 with all <0.001 of P-values (Figure 3B). These data indicate that the genetic alterations of HMGA2 concomitantly occurs with those of many TGF-β pathway core genes including PJA1/2 in HCC patients, suggesting that these concomitant genetic alterations of HMGA2 and TGF-β pathway core genes might be involved in tumorigenesis and development of HCC and thus, helpful as biomarkers for screening of HCC.

**Figure 4:**
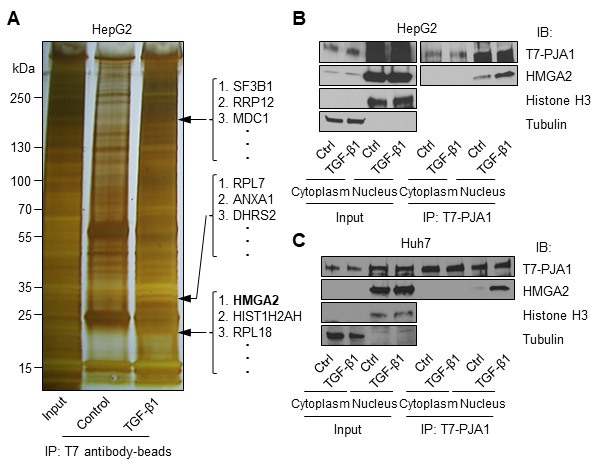
PJA1 interacts with HMGA2 in nuclear compartments of HCC cells. **A**. HepG2 cells were transfected with T7-PJA1 plasmid and treated with or without TGF-β1. The cell lysates were immunoprecipitated with T7 antibody-beads, loaded on SDS-PAGE gel, and silver-stained. Several bands that were observed in TGF-β1 treated lane, but not in the control lane-were dissected from the stained gel and analyzed by mass-spectrometry. The analysis identified the protein (arrow) as HMGA2. **B**. HepG2 and Huh7 cells were transfected with T7-PJA1 plasmid in 60 mm dishes and incubated in serum-free medium after one day. After 24 hours, the cells were treated TGF-β1 for 3 hours and the cell lysates were fractionated as described in the materials and methods. The fractionated lysates were immunoprecipitated with T7-antibody beads and immunoblotted with indicated antibodies.

Next, we explored proteins that interact with PJA1 in HCC cells through a TGF-β dependent manner. Co-immunoprecipitations with T7-beads and SDS-PAGE of HepG2 cell lysate with T7-PJA1 overexpression with or without TGF-β treatment were performed. After silver staining of the gel, we found three protein bands with molecular sizes of approximately 200, 30, and 20 kDa specific in the cell lysate of HepG2 treated with TGF-β (Figure 4A). Each band was found, by mass spectrometry analysis, to include several proteins. Among the candidates of PJA1 interacting proteins, HMGA2 can bind to SMAD3, and PJA1 can also bind to SMAD3. In addition, HMGA2 is induced by the TGF-β/SMAD pathway during EMT [24, 25]. Taken togethe, we have considered that PJA1 could interact with HMGA2.

To confirm the interaction between PJA1 and HMGA2, co-immunoprecipitation and Western blotting were performed using cell lysates after cell compartment fractionation in HepG2 and Huh7. The data showed that PJA1 interacted with HMGA2 in the nuclear fraction of both HepG2 and Huh7 cells and that treating the TGF-β for 3 hours enhanced the interaction between HepG2 and Huh7 cells (Figure 4B).

**Figure 5:**
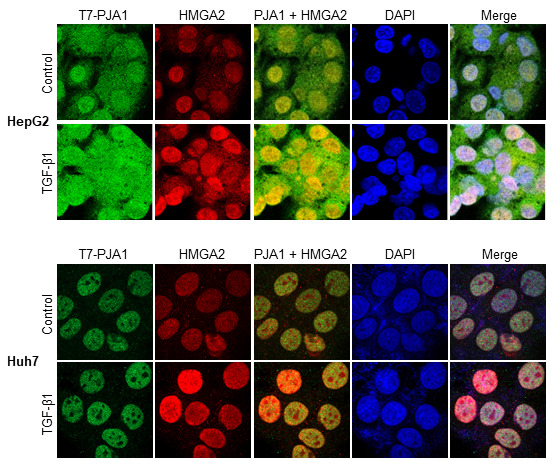
Confocal microscopic analysis showed that PJA1 colocalize with HMGA2 in the nuclei of HepG2 and Huh7 cells and TGF-β treatment enhances the colocalization. HepG2 and Huh7 cells were transfected with T7-PJA1 on 6 well plates with cover slips and incubated in serum-free medium after one day. After 24 hours, the cells were treated with TGF-β1 for 3 hours and fixed with 4% paraformaldehyde for 20 min. The fixed cells were premetallized with 0.1% Triton X-100, blocked with 10% normal goat serum for 30 min and incubated with T7 and HMGA2 antibodies for one hour. Then, the cells were labelled with goat anti-IgG antibodies (Green: Alexa Fluor 488, Red: Alexa Fluor 555) and counterstained with DAPI.

Next, confocal microscopy analysis was performed to investigate the co-localization of PJA1 and HMGA2 in HepG2 and Huh7 cells. The analyses demonstrated co-localization of PJA1 and HMGA2 in the nuclear of HepG2 and Huh7, and the co-localization was enhanced by TGF-β treatment for 3 hours (Figure 5).

## DISCUSSION

HMGA2 protein is overexpressed in many types of cancer including oral squamous cell carcinoma [27], lung cancer [28], breast cancer [29], gastric cancer [30], liver cancer [31], pancreatic cancer [32], colorectal cancer [33], ovarian cancer [34], and endometrial cancer [35]. Human telomerase reverse transcriptase (hTERT) is found to be essential for tumor cell proliferation and self-renewal properties [36, 37]. HMGA2 modulates hTERT transcription to promote tumorigenesis [38]. HMGA2 interferes with the recruitment of HDAC2 to the hTERT proximal promoter through interaction with transcription factor Sp1, leading to localized histone H3K9 hyperacetylation and thereby stimulating hTERT expression and telomerase activity. Interestingly, HMGA2 knocked-down by sh-HMGA2 in HepG2 cells leads to the progression of telomere shortening and a concurrent decrease of steady-state hTERT mRNA levels. As supported by this view, our recent TCGA analyses have demonstrated that both HMGA2 and TERT expression levels were raised in the alterations of the TGF-β pathway genes in 33 cancer types [10]. Although further study is necessary, the regulation of TERT by HMGA2 might play a role in cancers that have disrupted TGF-β pathways, including HCC.

HMGA2 is a non-histone chromatin-binding protein which contains three AT-hook domains that enable its binding to the minor groove of AT-rich DNA stretches [39, 40] and allows organized protein complexes on enhancers of various genes to regulate gene expression and cell differentiation. Although the function of the C-terminal acidic region is poorly understood, there are implications that C-terminal acidic tail in HMGA1 is involved in both the protein-protein interaction and the recruitment of factors during regulation of gene transcription [41]. We found that HMGA2 can interact with PJA1 in HCC cells, with the interaction increased due to TGF-β treatment. HMGA2 seems to undergo ubiquitination through PJA1 and/or some other E3 ligase; however, TGF-β does not potentiate the ubiquitination (data not shown). Further study is needed to determine whether the HMGA2 interacts with PJA1 through the C-terminal tail, and to determine if the HMGA2 /PJA1 complex that binds to DNA and HMGA2-target genes are regulated by the interaction with PJA1 in TGF-β signaling in HCC cells.

HMGA2 was identified as a prominent TGF-β target in TGF-β–induced EMT of murine mammary epithelial NMuMG cells by transcriptomic analysis and has been shown to be induced by the SMAD3 pathway during EMT [24, 42]. TGF-β induces the expression of HMGA2 by activating the transcription factor SMAD. HMGA2 associates with SMAD complexes to induce Snail and Twist expression, two established regulators of EMT, leading to mesenchymal transition [43, 44]. Interestingly, the overexpression of HMGA2 enhanced TGF-β signaling by activating expression of the TGF-β type II receptor that is localized to the invasive front of tumors [25]. In addition, the studies have shown that Smad3 and PJA1 respectively bind to HMGA2 and Smad3. These data led us to consider that PJA1 might interact with HMGA2. The TGF-β signaling pathway has been strongly implicated in EMT induction for many cell types [24, 25, 42, 45]. Our data clearly shows that PJA1 interacts with HMGA2 in the nucleus of HCC cells, suggesting that PJA1 might translationally control EMT of HCC in the TGF-β signaling pathway through the interaction with HMGA2.

Since it was also demonstrated that HMGA2 regulates the TGF-β signaling pathway, future research should be carried out to elucidate how the interaction between PKA1 and HMGA2 participates in TGF-β signaling pathways of HCC cells. The function of HMGA2 as an oncoprotein may be associated with several important molecules involved in EMT, invasion, and metastasis of HCC cells. These results further indicate that HMGA2 may serve as a potential target for the development of therapies for HCC, although additional detailed studies *in vivo* are required.

## MATERIALS AND METHODS

### Cell culture and transfection

HepG2 (HB8065) from ATCC and Huh7 (gift from Dr. Aiwu Ruth He’s lab, Georgetown University) were cultured in DMEM/F-12 medium and supplemented with 10% fetal bovine serum. HepG2 and Huh7 cells were transfected with T7-PJA1 plasmid using Lipofectamine LTX (Invitrogen) according to the manufacturer’s instruction. TGF-β1 (Sigma, T1654) was added to create a final concentration of 200 pM. Human PJA1 was purchased from GeneScript (OHu55728D) and was subcloned into pcDNA3.1 T7 plasmid.

### Mass-spectrometry analysis

HepG2 cells were transfected with T7-PJA1 plasmid and treated with or without TGF-β1 for three hours. The cell lysates were prepared with NP-40 buffer (50 mM Tris-HCl, pH 7.5, 0.15 M NaCl, 1% NP-40, 1 mM EDTA) with proteinase inhibitor cocktail (Roche Applied Science) and 1 mg of the proteins were immunoprecipitated with T7 antibody-beads. After washing with NP-40 buffer, the samples were denatured with 2x Laemmli sample buffer by heating and were loaded on 4-15% gradient SDS-PAGE gel and silver-stained (Pierce, Silver Stain for Mass Spectrometry, 24600). Bands that were observed in TGF-β1 treated lane, but not in the control lane, were dissected from the stained gel and sent to Harvard Medical School for mass-spectrometry analysis.

### Immunoblotting and immunoprecipitation analyses

Cells were lysed with lysis buffer (50 mM Tris-HCl, pH 7.5, 0.15 M NaCl, 1% NP-40, 1 mM EDTA), protease inhibitor cocktail (Roche Applied Science), 1 mM PMSF, 1 mM NaF, and 1 mM sodium orthovanadate. Nuclear and cytoplasmic proteins were prepared as follows: cells were harvested and incubated in buffer A (10 mM Hepes, pH 7.8, 10 mM KCl, 0.1 mM EDTA, 1 mM dithiothreitol, 2 mg/ml aprotinin, 0.5 mM phenylmethylsulfonyl fluoride, and 0.5% Triton X-100). After centrifugation, supernatants were collected as the cytoplasmic proteins. Buffer C (50 mM HEPES, pH 7.8, 420 mM KCl, 0.1 mM EDTA, 5 mM MgCl2, 10% glycerol, 1 mM dithiothreitol, 2 mg/ml aprotinin, and 0.5 mM phenylmethylsulfonyl fluoride) was added to the pellet. After rotation for 30 minutes and centrifugation, supernatants were collected as nuclear proteins. The following antibodies were used for immunoblotting and immunoprecipitation analyses: Flag-M2 (Sigma, F3165), Tubulin (T8328, Sigma), Histone H3 (sc-10809, Santa Cruz), T7 (A190-117A, Bethyl), HMGA2 (20795-I-AP, Proteintech), T7 Tag antibody agarose (69026, Novagen).

### Confocal microscopy analysis

For confocal imaging, cells were plated onto coverslips in 6-well plates. After TGF-β treatment, the cells were fixed with 4% paraformaldehyde, permeabilized in 0.1% Triton X-100, and blocked in 10% normal goat serum and PBS. The cells were incubated with primary antibodies, washed 3 times in PBS, and then incubated with goat anti-mouse or goat anti-rabbit secondary antibodies conjugated with Alexa-488 or Alexa-555 (Molecular Probes). 4’, 6-Diamidino-2-phenylindole (DAPI) was used for nuclear staining. The slides were then examined using a Zeiss LSM 710 or Zeiss spinning disk confocal microscope and the images were acquired with the Zen 2009 software.

## Abbreviations

HMGA2: High mobility group AT-hook 2
PJA: Praja ring finger E3 ubiquitin ligase
HCC: hepatocellular carcinoma
TGF-β: Transforming growth factor-β
TCGA: The Cancer Genome Atlas; PBS: Phosphate buffered saline
